# Prolactinomas in adolescent and elderly patients—A comparative long-term analysis

**DOI:** 10.3389/fsurg.2023.967407

**Published:** 2023-02-06

**Authors:** Lukas Andereggen, Angelo Tortora, Gerrit A. Schubert, Christian Musahl, Janine Frey, Markus M. Luedi, Luigi Mariani, Jürgen Beck, Emanuel Christ

**Affiliations:** ^1^ Department of Neurosurgery, Kantonsspital Aarau, Aarau, Switzerland; ^2^ Faculty of Medicine, University of Bern, Bern, Switzerland; ^3^ Department of Gynecology and Obstetrics, Kantonsspital Lucerne, Lucerne, Switzerland; ^4^ Department of Anaesthesiology and Pain Medicine, Inselspital, Bern University Hospital, University of Bern, Bern, Switzerland; ^5^ Department of Neurosurgery, University Hospital of Basel, Basel, Switzerland; ^6^ Department of Neurosurgery, Medical Center, University of Freiburg, Freiburg, Germany; ^7^ Department of Endocrinology, Diabetes and Metabolism, University Hospital of Basel, Basel, Switzerland

**Keywords:** prolactinoma, dopamine agonists, age, surgery, long-term outcome

## Abstract

**Objectives:**

Prolactinomas represent the most common type of secreting pituitary adenomas, yet are rarely encountered in adolescent-onset (AO; i.e. <18 years) or elderly-onset (EO; i.e. ≥65 years) cohorts. As a result, it is not clear whether long-term strategies should be focused differently at both age extremes when comparing their therapeutic outcomes. We aimed at investigating long-term endocrinological outcomes, looking for differences between the two cohorts and evaluating the dependence on continued dopamine agonist (DA) therapy.

**Methods:**

Retrospective cross-sectional comparative study analyzing prolactinoma patients with a follow-up of ≥4 years. Clinical, radiological and biochemical characteristics were assessed at diagnosis and last follow-up. Longitudinal endocrinological outcomes between groups of extreme ages (i.e. AO and EO) and middle age (i.e. ≥18 years to 65 years) were compared. Independent risk factors for long-term dependence on DAs were calculated.

**Results:**

Follow-up at ≥4 years was recorded for 108 prolactinoma patients; 10 patients with AO and 10 patients with EO. Compared to AO patients, EO patients were predominantly men (*p* = 0.003), and presented with significantly higher prolactin (PRL) levels (*p* = 0.05) and higher body mass index (*p* = 0.03). We noted a significant positive correlation between patients' PRL values and their age (r = 0.5, *p* = 0.03) or BMI (*r* = 0.6, *p* = 0.03). After a median follow-up of 115 months, remission was noted in 87 (83%) patients; 9 (90%) in AO patients, and 7 (70%) in EO patients (*p* = 0.58). Continuation of DAs was required in 4 patients (40%) with AO and 7 patients (70%) with EO (*p* = 0.37). Patients with elderly-onset were an independent predictor of long-term dependence on DAs (HR 2.8, 95% CI 1.1-7.2, *p* = 0.03).

**Conclusions:**

Long-term control of hyperprolactinemia and hypogonadism does not differ between members of the AO and EO cohorts, and can be attained by the majority of patients. However, adjuvant DAs are often required, independent of the age of onset. Considering the clinical significance of persistent DA therapy for the control of hyperprolactinemia in many patients at both extremes of age, long-term monitoring may become recommended, in particular in patients with elderly-onset.

## Introduction

While prolactinomas account for 32% to 66% of all pituitary adenomas (1), they are rarely encountered in adolescent (AO) or elderly (EO) patients (2–6). Although the incidence of pituitary adenomas increases with age (7, 8), data on prolactinoma patients who are elderly at onset are infrequently reported, probably because endocrinological and neurological symptoms get misinterpreted as age-related disturbances (9). On the other hand, the prevalence of dopamine-agonist (DA) treated hyperprolactinemia has a preponderance in women with a peak at 25–34 years (10), and a much later peak in men (11). However, many symptoms of pituitary adenoma reported in adulthood were already evident during adolescence, suggesting that the true prevalence of AO's is higher than initially believed (2). As a result, it is not clear whether long-term strategies should be focused differently at both age extremes when comparing the therapeutic long-term outcomes.

We aimed at investigating long-term endocrinological outcomes, including differences between the adolescent and elderly cohorts and assessment of the dependence on continued DA therapy.

## Methods

### Study design

We analyzed the medical data of a prospectively maintained database, including all consecutive prolactinoma patients with middle age onset (i.e., ≥18 years to 65 years), and age limits defined as AO (≤18 years), EO (≥65 years) treated from January 1997 to December 2015. The minimum follow-up period permitting documentation of longitudinal changes was set at ≥4 years. Patients' demographics and endocrinological characteristics at baseline and last follow-up were analyzed. The Human Research Ethics Committee of Bern (Cantonal ethical commission KEK Bern, Bern, Switzerland) approved the study (KEK n° 10-10-2006 and 8-11-2006).

### Clinical and biochemical assessment

Diagnosis was based on clinical and biochemical assessment, including a standard protocol for pituitary magnetic resonance imaging (MRI; see below). All patients fulfilled the diagnostic criteria of a prolactin (PRL)-secreting pituitary adenoma [i.e., elevated PRL levels without evidence of pituitary stalk compression, primary hypothyroidism or drug-induced hyperprolactinaemia, and positive pituitary magnetic resonance imaging (MRI) scan] (12, 13). Prolactin (PRL) levels, including the immunoradiometric PRL assay with serum dilution in order to overcome the high-dose PRL hook effect (14), were measured. The upper limits of PRL levels for diagnosis were set at 20 µg/L(15). Partial hypopituitarism was defined as impaired secretion of one or more pituitary hormones. Secondary adrenal insufficiency was characterized by the presence of low serum cortisol (<50 nmol/L) levels, or normal cortisol but inadequate responses to the adrenocorticotropin (ACTH) stimulation test or insulin tolerance test. The diagnosis of secondary hypothyroidism was made based on a finding of low-normal thyroid-stimulating hormone (TSH) levels and a low free thyroxin (FT4) level. A gonadotropin deficiency or central hypogonadism was considered in the case of low-normal levels of gonadotropins in parallel with low estradiol/testosterone levels.

### Assessment of BMI

A standard body mass index (BMI) was calculated for all patients (16). A BMI of 21-25 kg/m^2^ was defined as normal, BMI 26–30 kg/m^2^ as overweight, BMI 31–35 kg/m^2^ as obese, and BMI > 35 kg/m^2^ as severely obese.

### Radiological assessment

MRI was performed on a 1.5- or 3-Tesla system including a Proton/T2-weighted whole-brain study with unenhanced, contrast-enhanced, dynamic contrast-enhanced and post contrast-enhanced overlapping studies over the sellar region (17–20). A tumor with a diameter of 1–10 mm was defined as a microadenoma, and >10 mm as a macroadenoma. Infiltration of the cavernous sinus was noted (i.e., Knosp grade ≥1) (21, 22).

### Indication for surgery

Besides local prolactinoma characteristics, such as apoplexy with visual disturbances or cystic adenomas, the indication for surgery was discussed by an interdisciplinary team, including the patient, and the decision was based on the patient's preference for surgical treatment rather than long-term DA therapy (23, 24). Pituitary surgery was performed using a transseptal, transsphenoidal microsurgical approach (i.e., transsphenoidal surgery, TSS) with standardized sellar reconstruction, as previously described (17–20).

### Long-term assessment

A standardized protocol was followed for withdrawal from the use of DAs over the long term (25). If PRL levels had normalized and tumor reduction of >50% was attained, DAs were tapered 24 months after initiation of the medical therapy (26, 27). Recurrence was defined as an increase in PRL levels above the normal range (>20 µg/L) during the last follow-up period after a previous remission, irrespective of the radiological findings (28, 29).

### Statistical analysis

Data were analyzed using IBM SPSS statistical software Version 24.0 (IBM Corp., New York, NY, USA) and GraphPad Prism (V7.04 software, San Diego, CA, USA). Continuous variables were examined for homogeneity of variance and are expressed as mean ± SD unless otherwise noted. Serum PRL levels are presented as median values and interquartile range (IQR, 25th to 75th percentile). Categorical variables are given as numbers and percentages. For comparisons of means between groups (i.e., patients with AO and EO), Student's t-test was used for normally distributed data, and the Mann–Whitney test for nonparametric data. The Wilcoxon signed-rank test was used to evaluate paired differences in PRL and BMI levels before and after treatment. Categorical variables were compared using Pearson's chi-square test or Fisher's exact test, as appropriate. The Spearman rank-order correlation coefficient was calculated to check for the strength of association between different variables (i.e., PRL, age, patients' BMI, DA dependency). We assessed the proportion of patients with long-term dependence on DAs and performed time-dependent multivariable regression analysis to calculate hazard ratios (HR) for potential risk factors. The variables tested were: age at diagnosis, initial PRL levels, BMI (kg/m^2^), hypopituitarism at diagnosis, baseline gonadotropin deficiency, prevalence of headache at diagnosis, adenoma size, and cavernous sinus invasion. The multivariable regression analysis included all dependent risk factors in the univariable regression with a *p* value ≤ 0.05. Baseline PRL values were log transformed before being imputed in the regression and correlation analysis analysis, as data showed a positively skewed distribution. Significance level was set at *p* ≤ 5%.

## Results

### Patients' characteristics at diagnosis

**Figure 1:**
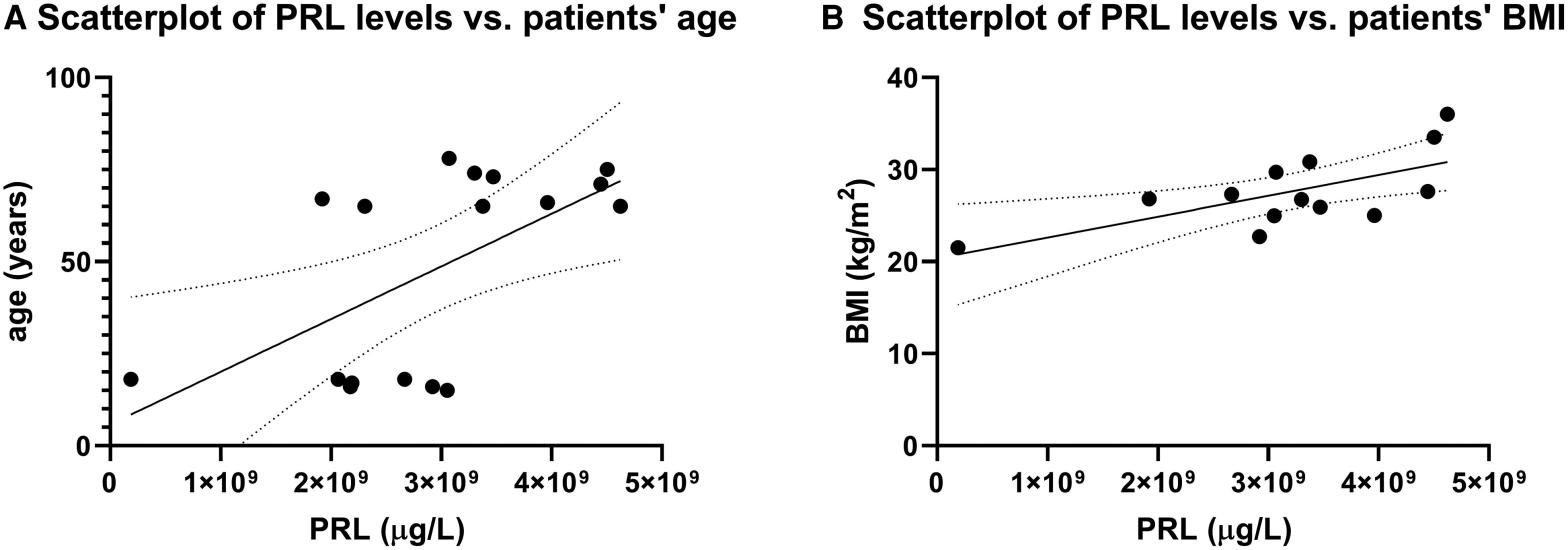
Correlation between BMI and PRL levels. (**A**) Scatterplots reveal a significant positive correlation between patients’ serum baseline PRL values and age (*r* = 0.5, *p* = 0.03). Likewise, we noted a significant positive correlation between baseline PRL values and patients’ BMI (*r* = 0.6, *p* = 0.03).

A follow-up at ≥4 years was performed for 108 patients; 10 women with AO, and 3 women and 7 men with EO. Baseline characteristics are detailed in Table 1, Table 4. At diagnosis, mean age was 16.8 years (range 14–18) in the AO cohort and 69.9 years (range 65–78) in the EO cohort. Male sex, baseline PRL levels, and patients’ BMI were significantly higher in the EO cohort. The prevalence of macroadenomas or adenomas with infiltration of the cavernous sinus (i.e., Knosp grade ≥1) was higher in the EO than the AO cohort, though the results were not statistically significant. First-line TSS was performed in 63 (58%) patients   . In patients at extreme ages, we noted a significant positive correlation between patients' PRL values, and both their age (*r* = 0.5, *p* = 0.03, Figure 1A), and their BMI values (*r* = 0.6, *p* = 0.03, Figure 1B), respectively.

**Table 1:** Patient characteristics at baseline.

Patients’ characteristics	<18 years	>65 years	≥18 yrs–≤65 yrs	All patients
Number of patients, *n* (%)	10 (9)	10 (9)	88 (82)	108 (100)
Age (years), mean ± SD	16.8 ± 1.5 (14–18)	70 ± 5.4 (65–78)	37 ± 11 (21–62)	38 ± 15 (14–78)
Sex: women, *n* (%)	10 (100)	3 (30)	67 (76)	80 (74)
BMI (kg/m^2^), mean ± SD	24.1 ± 2.6 (22–27)	29.1 ± 3.7 (25–36)	26.3 ± 5.3 (18–43)	26.6 ± 5.1 (18–43)
PRL levels, median (IQR)	155 (117–833)	2,665 (935–28,985)	212 (108–823)	227 (116–1093)
Macroadenoma, *n* (%)	5 (50)	9 (90)	46 (43)	60 (56)
Cavernous sinus infiltration, *n* (%)	3 (30)	6 (60)	32 (36)	41 (38)
TSS, *n* (%)	5 (50)	1 (10)	57 (65)	63 (58)
Hypopituitarism, *n* (%) Affected pituitary axes, *n* (%)	7 (70)	9 (90)	64 (76)	80 (77)
Gonadotropin deficiency	7 (70)	9 (90)	64 (80)	80 (80)
Secondary hypothyroidism	0 (0)	3 (30)	4 (5)	7 (7)
Secondary adrenal insufficiency	0 (0)	2 (20)	3 (4)	5 (5)
Headache, *n* (%)	2 (22)	5 (50)	17 (20)	24 (23)

PRL, prolactin (µg/L); IQR, interquartile range; *n*, numbers; BMI, body mass index; SD, standard deviation.

### Characteristics at last follow-up

**Figure 2:**
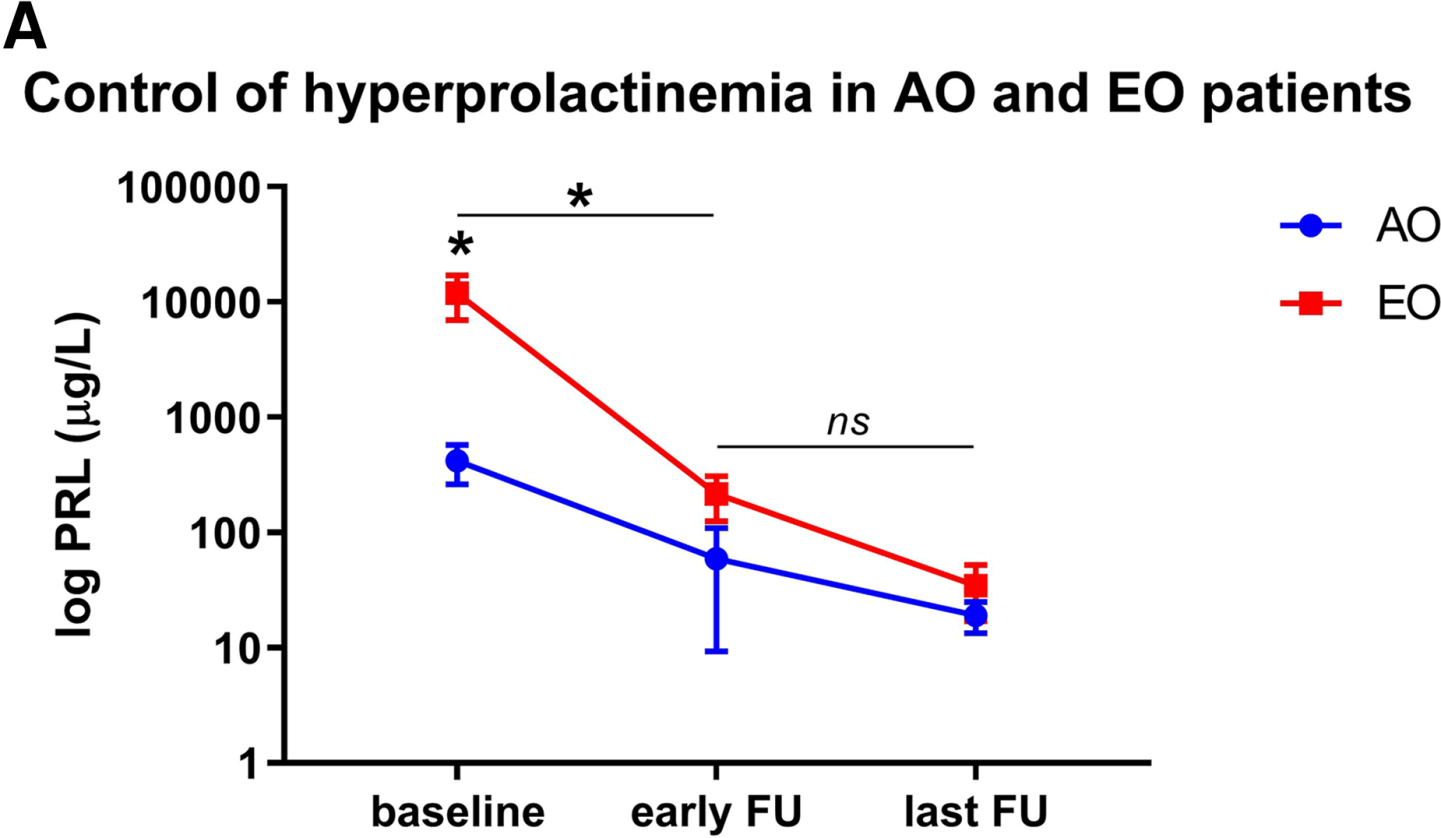
PRL levels in adolescent-onset (AO) patients and elderly onset (EO) patients. (**A**) Differences in PRL levels in both cohorts at baseline, early (i.e. 3 months) and last follow-up. Baseline PRL levels were significantly higher in patients in the EO cohort than the AO cohort (*p* = 0.05), but not at early (*p* = 0.20) or long-term follow-up (*p* = 0.39). PRL levels at early follow-up significantly decreased in both cohorts compared to baseline values (*p* = 0.04 both in the AO and EO cohort). There is no significant difference between early and long-term PRL values (*p* = 0.48 for AO; *p* = 0.07 for EO, respectively).

Patients' characteristics at last follow-up are detailed in Table 2, Table 5. The median follow-up period was 115 months (range, 48–408 months). PRL levels decreased from 155 µg/L (IQR 177–833 µg/L) to 14 µg/L (IQR 9–24 µg/L; *p* = 0.06) in the AO cohort, and from 2665 µg/l (IQR 935-28985 µg/L) to 15 µg/L (IQR 9-37 µg/L; *p* = 0.04; Figure 2) in the EO cohort, respectively. Serum PRL levels at last follow-up were not significantly different between the groups at extreme ages (*p* = 0.42).

**Table 2:** Patient characteristics at long-term follow-up.

Patients’ characteristics	<18 years	>65 years	≥18 yrs–≤65 yrs	All patients
BMI (kg/m^2^), mean ± SD (range)	28.0 ± 6.7 (18–38)	29.5 ± 5.1 (25–40)	26.4 ± 5.2 (17–42)	26.8 ± 5.3 (17–42)
PRL levels, median (IQR)	14.4 (9.2–23.8)	15 (9.2–37.7)	14.1 (7.7–22.4)	14.4 (7.7–22.6)
Remission, *n* (%)	9 (90)	7 (70)	71 (84)	87 (83)
DA required, *n* (%)	4 (40)	7 (70)	50 (57)	62 (57)
Hypopituitarism, *n* (%)	1 (10)	5 (50)	25 (28)	31 (29)
Affected pituitary axes, *n* (%)				
Gonadotropin deficiency	0 (0)	2 (20)	16 (18)	18 (17)
Secondary hypothyroidism	0 (0)	2 (20)	8 (9)	10 (9)
Secondary adrenal insufficiency	1 (10)	3 (30)	7 (8)	11 (10)
Headache, *n* (%)	1 (10)	0 (0)	1 (1)	2 (2)
Follow-up time in months, median (range)	114 (65–264)	56 (48–174)	112 (48–408)	115 (48–408)

PRL, prolactin (µg/L); IQR, interquartile range; *n*, numbers; BMI, body mass index; SD, standard deviation.

There was an increase in AO patients' BMIs over the long-term, from 23.8 ± 2.6 kg/m^2^ to 26.9 ± 6.7 kg/m^2^; *p* = 0.46), but it was not statistically significant. Obesity (BMI > 31 kg/m^2^) was noted in 3 (30%) AO patients and 2 (20%) EO patients. The rate of gonadotropin deficiency significantly decreased in both cohorts at extreme ages (*p* = 0.003 for AO and *p* = 0.006 for EO). Over the long-term, there was no significant difference in the rates of gonadotropic, thyrotropic or corticotropic insufficiencies. Remission was noted in 87 (83%) patients, including 9 (90%) with AO and 7 (70%) with EO (*p* = 0.58). For the long-term control of hyperprolactinemia, a greater, but non-significant need for continuation of DAs was noted in 70% of EO patients compared to 40% of AO patients (*p* = 0.37).

The risk factors for long-term DA dependence are summarized in Table 3. Significant risk factors in the univariable analysis included patients with EO, baseline PRL levels, hypopituitarism at diagnosis, presence of a macroprolactinoma, and cavernous sinus invasion. Multivariable Cox regression analyses revealed patients with EO as an independent risk factor for dependence on DAs (HR 2.8, 95% CI 1.1–7.2, *p* = 0.03).

**Table 3:** Predictors of long- term dependence on dopamine agonists.

Predictors of long-term DA dependence	Univariable analyses HR (95% CI)	*p*-value	Multivariable analyses HR (95% CI)	*p*-value
Age (i.e. EO)	3.9 (1.8–8.3)	<0.001	2.8 (1.1–7.2)	0.03
Baseline PRL levels (ug/L)	1.6 (1.2–2.3)	0.01	0.9 (0.4–1.6)	0.61
Baseline BMI (kg/m^2^)	1.1 (1.0–1.1)	0.08		
Baseline hypopituitarism	2.3 (1.1–4.6)	0.02	1.6 (0.5–-5.0)	0.39
Baseline gonadotropin deficiency	1.9 (0.9–4.3)	0.1		
Headache	1.7 (0.9–3.0)	0.09		
Adenoma size (i.e. Macroadenoma)	2.1 (1.2–3.5)	0.01	0.9 (0.3–2.6)	0.91
Cavernous sinus invasion	2.4 (1.5–4.0)	0.001	2.2 (0.8–5.9)	0.11

BMI, body mass index; CI, confidence intervals; DA, dopamine agonist; HR, hazard ratio; PRL, prolactin.

## Discussion

Our long-term data indicate that at a median follow-up of 9.5 years, control of hyperprolactinemia and hypogonadism does not differ between members of the AO and EO cohorts, and can be attained by the majority of patients. However, adjuvant DAs are often required, in particular in patients with elderly-onset.

Although prolactinomas represent the most common type of secreting pituitary adenomas, their diagnosis both in adolescents and in the elderly is rare (30). Pituitary adenomas in young patients typically present with endocrinopathies in keeping with their adenoma type, rather than resulting in local complications, meaning that macroadenomas are less frequently encountered (31). In accordance with these findings, we noted that macroprolactinomas were present in 90% of patients in the EO cohort vs. 50% of patients in the AO cohort. Likewise, the number of female patients was significantly higher in the AO group than the EO group. This corroborates a report on 41 patients ≤21 years of age, in which the disruption of the menstrual cycle was the most common symptom encountered in 85% of female patients, with the majority being diagnosed with a microprolactinoma (31).

While amenorrhea in female patients is clinically apparent and easily detected, non-specific symptoms of hypogonadism in men, such as loss of libido, are frequently not reported. Subsequently, a higher prevalence of macroprolactinomas in men than in women has been recorded (32, 33). As macroprolactinomas are associated with longer lasting hyperprolactinemia and related hypogonadism (34, 35), the significantly higher rates of baseline PRL levels in the EO cohort probably reflect on the longer disease duration in more oligosymptomatic men (36, 37). Likewise, EO was an independent predictor of long- term dependence on DAs. This is an intriguing finding. In a recent meta-analysis, the probability of persistent hyperprolactinemia was higher in younger patients (38). However, their participant mean cut-off age was set at 33.2 years, which is considerably lower than the reported age groups of the present analysis (i.e., EO), where the disease is extremely rare. As hyperprolactinaemia was showed to recur early in most macroprolactinomas (93%) following DA therapy discontinuation after 7 years of therapy (39), or in those adenomas with cavernous sinus infiltration (40), it is conceivable that the greater number of patients with a macroprolactinoma and those with cavernous sinus infiltration in the EO cohort allegedly overlooks a significant effect given the relatively small number of patients fulfilling the study inclusion criteria. Beside the 70% patients in the EO cohort with continuing DA therapy, it is striking that 40% of patients in the AO cohort were still dependent on DAs after almost 10 years too. These results corroborates with a recent review reporting of less than half of pediatric patients with prolactinomas were eligible for DA withdrawal, and less than one-fourth achieved control of hyperprolactinemia following treatment cessation (41). Both the Pituitary (42) and Endocrine Society (15) recommends tapering DAs after three and two years of treatment in the case of PRL normalization, respectively. However, in contrast to initial studies reporting that many patients treated with DAs remained in remission after drug withdrawal (27), frequent early recurrence of hyperprolactinemia following discontinuation of DAs is becoming increasingly common (43, 44). In addition, cumulative doses over the long term might contribute to potentially adverse effects, including the recently documented personality changes (45), lack of compliance, and inconvenience for patients (46, 47). As for controlling hyperprolactinemia and associated hypogonadism, both DAs and surgery can be effective in selected patients (1, 25, 36, 48–50).

With the goal of minimizing the need for continuation of DAs over the long term, upfront surgery has been proposed in highly selected patients (49, 51, 52). Although surgery might be effective with regard to non-dependency on DA therapy in the long-term, particularly in microprolactinomas (51, 52) or macroprolactinomas not infiltrating the cavernous sinus (49), results are mixed. Ongoing long-term DA therapy after surgery has been reported in 66% patients with prolactinomas (53). In addition, treatment guidelines focusing on long-term strategies becomes vague in patients at extreme ages. While the prevalence of DA treated hyperprolactinemia has a preponderance in women with a peak at 25–34 years (10), it has been suggested that children and adolescents with a prolactinoma should receive DAs as a first-line treatment (31, 42). However, normalization of hyperprolactinemia frequently requires DA over the long term (22). As a result, TSS was predominantly the treatment of choice in the AO onset compared to the EO, though not significantly. Independent of the selected first-line treatment, long-term monitoring of patients with prolactinomas thus becomes necessary, highlighting the need for a longitudinal survey in young and – considering the risk of persistent DA therapy – in old cohorts in particular.

Interestingly, we noted a significant correlation between baseline PRL levels and both patients' BMI and age, along with higher baseline PRL and BMI values in the EO compared to the AO cohort. While it is conceivable that increases in BMI are partly related to the normal age-associated increase in body weight, hyperprolactinemia and associated hypogonadism have been shown to impact patients' BMIs (54). While the underlying mechanism remains unclear (55), this association is possibly related to the longer exposure to increased PRL levels and associated hypogonadism in elderly patients and/or men with non-specific symptoms (56). Of note, BMI values over the long term were not significantly different in the two cohorts, with a non-significant increase in BMI values being noted in the AO cohort despite control of hyperprolactinemia in the majority of group members. In a study of young patients with pituitary adenomas, the highest BMI at diagnosis was measured in prolactinoma patients, with ongoing weight gain in some patients despite normalization of PRL levels (31). While several studies reported on weight loss following control of hyperprolactinemia (57), others couldn't confirm this association (58). Thus, control of hyperprolactinemia and hypogonadism remains the primary treatment target, and a weight control program may be necessary, as obesity resulting from pituitary adenomas can lead to significant morbidity and mortality (54).

## Study limitations

The main limitations of our study are the small sample size, and its retrospective, single-center design. To allow for statistical comparison between the AO and EO cohorts, we calculated the BMI in all patients. Given that the lower age range in the AO cohort was 14 years, we used BMI z-scores or BMI standard deviation scores (SDS) to calculate the relative weight adjustment for a child's age and sex (59). Despite the relatively small number of patients meeting the inclusion criteria, we believe this study adds to the existing literature, presenting important changes in longitudinal outcome in patients with adolescent- and elderly-onset followed for up to 22 years. Our findings could have with direct implications for the care of patients with prolactinomas at extreme ages.

## Conclusions

Long-term control of hyperprolactinemia and hypogonadism does not differ between members of the AO and EO cohorts, and can be attained by the majority of patients. Considering the clinical significance of persistent DA therapy for the control of hyperprolactinemia in many patients at both extremes of age, long-term monitoring may become recommended, in particular in patients with elderly-onset.

## Data Availability

The original contributions presented in the study are included in the article/Supplementary Material, further inquiries can be directed to the corresponding author/s.
